# Supplementary material for: Content-rich biological network constructed by mining PubMed abstracts
Source: BMC Bioinformatics. 2004 Oct 8;5:147. doi: 10.1186/1471-2105-5-147 (PMC528731; doi:10.1186/1471-2105-5-147)
Supplement: Additional File 5 — The original Chilibot query results of the term "long-term potentiation (LTP)" and 22 other terms, limiting the latest references analyzed to the years 1990, 1995, 2000, and 2004. [file 1471-2105-5-147-S5.bz2 › chilibotAdditionalFile5/ltp2004/html/CAMKIV.html]

 


**CAMKIV** (Input: CAMKIV ) 

---


**Google Searches:** Entire Web  | EDU domain only  | PDF files only

.

**External Links:** OMIM |
LocusLink
| Swissprot
| GeneCards

  
**Maps of CAMKIV**

Simple
Complete
graph in
radiant
tree
square
layout.

**New Hypothesis !**

**Synonyms** 

- camkiv   [PubMed]

**Synopsis**

- The results suggest that CaMKII is involved in LTP induction, while **CaMKIV** and MAPK are rather involved in LTP maintenance.  Nippon Yakurigaku Zasshi, 2002 
  [23]

- Our results suggest that **CaMKIV** functions as a CREB kinase and controls CREB regulated gene expression during HFS induced LTP in the rat hippocampal CA1 region.  J Biol Chem, 2001 
  [23]

- These results indicate that neural activity dependent **CaMKIV** signaling in the neuronal nucleus plays an important role in the consolidation retention of hippocampus dependent long term memory.  Cell, 2001 
  [23]

- These results support an important role for **CaMKIV** Gr in calcium regulated neuronal gene transcription and synaptic plasticity and suggest that the contribution of other signaling pathways may spare spatial memory of **CaMKIV** Gr deficient mice.  J Neurosci, 2000 
  [20]

- Thus, in addition to defining an important signaling role for **CaMKIV** in a subpopulation of T cells, we identify differential signaling requirements for cytokine production between naive T cells and T cells that express cell surface markers characteristic of the memory phenotype.  Mol Cell Biol, 2002 
  [16]

- Notably, CD28 ligation activated p38 MAPK and **CaMKIV**, the kinases stimulated by CD3 engagement, suggesting that CD28 acts by increasing the activation extent of p38 MAPK and **CaMKIV**.  J Immunol, 2001 
  [16]

- We have found that in **CaMKIV** mice which are deficient in a component of the calcium calmodulin dependent protein kinase CaMK pathway, a major pathway of CREB activation fear memory,   Nat Neurosci, 2002 
  [16]

- These results indicate that the activation of **CaMKIV** and CREB are necessary to establish a late phase of cerebellar LTD.  Neuron, 1999 
  [14]

- Our results provide genetic evidence that TrkB mediates hippocampal plasticity via recruitment of PLCgamma, and by subsequent phosphorylation of **CaMKIV** and CREB.  Neuron, 2002 
  [14]

- These data are compatible with a model where calcium influx via L type calcium channels prevents caspase dependent cleavage of **CaMKIV** and promotes neuronal survival by maintaining a constitutive level of **CaMKIV** CREB dependent gene expression.  FASEB J, 2001 
  [11]

- Activation is classically brought about by signaling dependent phosphorylation of a key acceptor site by a number of possible kinases, including PKA, **CamKIV**, and Rsk 2.  Exp Cell Res, 2002 
  [10]

- CaM binding proteins CaMBPs as Ras GRF and CaM dependent protein kinase IV **CaMKIV** positively modulate ERK1 2 activation induced by either NGF or membrane depolarisation in neurones.  Cell Signal, 2002 
  [10]

- The focus of this review is to describe the biochemical regulation of **CaMKIV** and examine how **CaMKIV** activates transcription in response to calcium in both cell and animal models.  Biometals, 1998 
  [10]

- the **CaMKIV** pathway may be well suited to conveying information on the precise timing of localized synaptic stimuli, befitting its greater speed and sensitivity.  Proc Natl Acad Sci U S A, 2001 
  [10]

- We propose that sequestration of CaMKK may be the molecular mechanism by which catalytically inactive mutants of **CaMKIV** exert their dominant negative functions within the cell.  J Biol ChemJ Biol Chem, 2001 
  [10]
